# UV-assisted fluctuation-enhanced gas sensing by ink-printed MoS_2_ devices

**DOI:** 10.1038/s41598-024-73525-2

**Published:** 2024-09-27

**Authors:** Katarzyna Drozdowska, Janusz Smulko, Jakub Czubek, Sergey Rumyantsev, Andrzej Kwiatkowski

**Affiliations:** 1 Department of Metrology and Optoelectronics, Faculty of Electronics, Telecommunications, and Informatics, Gdańsk University of Technology, G. Narutowicza 11/12, 80-233 Gdańsk, Poland; 2 Institute of High Pressure Physics PAS, CENTERA Laboratories, Warsaw, Poland

**Keywords:** Electrical and electronic engineering, Sensors and biosensors

## Abstract

In this work, MoS_2_ flakes were printed on ceramic substrates and investigated toward 1–10 ppm of nitrogen dioxide (NO_2_), 2–12 ppm of ammonia (NH_3_), and 2–12 ppm acetone (C_3_H_6_O) under UV light (275 nm). The structure of overlapping MoS_2_ flakes and UV light assistance affected high responsivity to NO_2_ when DC resistance was monitored, and superior sensitivity to NH_3_ was obtained from the low-frequency noise spectra. MoS_2_ exhibited response and recovery times in hundreds of seconds and stability throughout the experiments conducted within a few months. MoS_2_ sensor exhibited a resistance drift during the detection of a specific relaxation time. Subtracting the baseline burden with exponential drift exposed the direction of changes induced by oxidizing and reducing gases and reduced DL to 80 ppb, 130 ppb, and 360 ppb for NO_2_, NH_3_, and C_3_H_6_O, respectively. The fluctuation-enhanced sensing (FES) revealed that the adsorption of NO_2_ on MoS_2_ decreases the noise intensity, whereas adsorbed NH_3_ increases the fluctuations of current flowing through the sensor, and these changes are proportional to the concentration of gases. The noise responses for NO_2_ and NH_3_ were opposite and higher than DC resistance responses with subtracted baseline (an increase of 50% for 10 ppm of NO_2_ and an increase of more than 600% for 12 ppm of NH_3_), showing that FES is a highly sensitive tool to detect and distinguish between these two gases. This way, we introduce a simple and low-cost method of gas sensor fabrication using ink-printed MoS_2_ and the possibility of enhancing its sensitivity through data processing and the FES method.

**Supplementary Information:**

The online version contains supplementary material available at 10.1038/s41598-024-73525-2.

## Introduction

The unique electronic, optical, and structural properties of various two-dimensional (2D) materials are attractive to researchers from different fields of science and explored for manufacturing field-effect transistors (FETs), solar cells, supercapacitors, and photodetectors^1^. The structure of bulk MoS_2_ consists of 2D S-Mo-S layers attracted to each other by weak van der Waals forces. Due to this fact, similarly to graphene, MoS_2_ can be easily exfoliated to its 2D form. 2D MoS_2_ usually exhibits *n*-type conductivity; however, depending on the synthesis process or if it forms a junction with other materials, it can also act as a *p*-doped semiconductor^2,3^. Monolayered MoS_2_ is characterized by attractive structural and transport properties, including large specific surface area and surface activity, as well as high carrier mobility, which is beneficial compared to other gas-sensitive materials such as pure metal oxides or polymers. At the same time, gas sensors based on the pristine form of this material can exhibit limited selectivity and extended sensing response and recovery time, which is unfavorable in gas detection and still requires fixing^4^. Still, the optoelectronic properties of MoS_2_ attract great attention for light-assisted gas sensing, as the bandgap of MoS_2_ (and other TMDCs as well) depends on a number of monolayers and enables tuning of their electrical properties with light, which is not possible in graphene^5^. The monolayered MoS_2_ has a direct bandgap of ~ 1.8–1.9 eV, which falls in the red-light range (~ 660–690 nm). Because of that, some reports on light-assisted gas sensing by monolayered MoS_2_ focus on detection enhanced by red light, which generates extra charge carriers participating in molecular adsorption^6^. The mechanisms of gas adsorption/desorption by mono- or a few-layered MoS_2_ confirmed by theoretical computations and experimental works are well summarized elsewhere^7,8^. In the case of organic vapors, their detection is limited due to the poor carrier exchange between the molecules and the sensing layer. Thus, e.g., sensing traces of acetone (sub-ppm concentrations) usually requires elevated operating temperatures, which also facilitates detection in humid conditions^9–11^.

The gas-sensing properties of MoS_2_ can be enhanced by different means, mainly to improve the sensor response and recovery times, sensitivity, and selectivity^12,13^. Doping with noble metals or metal oxides^2,14^, forming a junction with other nanomaterials^15^, elevated temperatures, and visible or UV light assistance^6,16^ were tested by numerous research groups. For instance, selectivity to NO_2_ was obtained when MoS_2_ was irradiated with UV light^17^. However, the method of fluctuation-enhanced sensing (monitoring low-frequency noise spectra) was also utilized to increase the selectivity of MoS_2_-based sensors to organic vapors such as chloroform, tetrahydrofuran, acetonitrile, and acetone^18^. The random resistance component can be highly informative about the surface processes of gas adsorption. Fluctuation-enhanced sensing (FES) utilizes low-frequency noise spectra as a sensitive tool for gas detection. The flicker noise (1/*f* noise) can be affected by molecular adsorption due to the transfer of charges, resulting in fluctuations in their concentration and mobility^19^. The adsorption of molecules can result in a change in the noise intensity but also in the shape of the spectra (change in the slope due to induction of dominant generation-recombination events that produce the Lorentzians of characteristic frequencies). Until now, FES was successfully applied to distinguish between organic vapors by graphene back-gated FET or to determine the concentration of NO_2_ by networks of carbon nanotubes^20,21^. Thus, the method can potentially be a highly sensitive tool for devices based on other low-dimensional materials, including MoS_2_. Since FES has not been applied to layers of 2D semiconducting flakes, our studies fill a research gap for energy-efficient gas sensors with high potential for practical applications^22–24^.

Since high-performance room-temperature gas detection remains an open issue for devices based on low-scale structures, light-activated sensors attract great attention. On the one hand, quanta of the visible light carry enough energy to generate electron-hole pairs and induce photoconductivity^25^. Increasing the current flowing through the sensor is essential for any practical applications since MoS_2_ layers are usually of high resistance. On the other hand, UV light activates the material surface by partly desorbing oxygen species and humidity and creating weakly bonded oxygen ions (oxygen photoions) that can be easily replaced by target gas molecules during detection^26^, so it can stabilize and enhance sensing responses and secure photodesorption during recovery by shortening its time.

For fabricating sensors that rely on 2D materials, additive manufacturing has recently become an intriguing approach^27,28^. In the case of printed MoS_2_-based gas sensors, Yao et al. showed promising sensitivity toward 5 ppm of NH_3_ at room temperature in 2013^29^. Simultaneously, the authors highlighted that the sensor needs further optimization due to the long response and recovery times. In the case of NO_2_ and NH_3_ sensing shown by Chen et al.^30^, the detection limit in the ppb range was obtained for a single MoS_2_ flake, but at 100 V bias. Thus, reducing the energy consumption for printed MoS_2_-based sensor operation still requires investigation. Nevertheless, the easy and low-cost fabrication of printed MoS_2_ and its attractive optoelectronic properties hold a promise for high-quality performance as light-enhanced gas sensors. This approach solves the practical problem of proposing low-cost gas sensors of reduced energy consumption.

Herein, we studied the optical properties of the MoS_2_ flakes dispersion and MoS_2_-based sensor responses toward three gases, representing different types of species and interactions with low-dimensional MoS_2_, namely nitrogen dioxide (NO_2_), ammonia (NH_3_), and acetone (C_3_H_6_O). We aim to propose a simple fabrication method of producing MoS_2_-based sensors and post-processing the obtained responsivity data to enhance the sensitivity and provide sensors of low detection limits at RT. We also utilize the FES method and show that the intensity of fluctuations generated in the MoS_2_ sensor depends on the type and concentration of the selected gases, which increases the selectivity of such sensors. This way, we point toward a reproducible and easy method of producing and measuring gas sensing devices based on 2D materials and sensing data analysis.

## Methods

### MoS_2_ sensors fabrication

MoS_2_ flakes dispersed in ethanol-water solution (flakes concentration of 18 mg/L) were purchased from Graphene Supermarket. According to the producer, MoS_2_ flakes are of 100–400 nm lateral size and thickness of 1–8 monolayers. The structural and optical characteristics of MoS_2_ flakes, including SEM and AFM images, UV-Vis absorption, and Raman spectra, can be found on the producer’s website (www.graphene-supermarket.com). Al_2_O_3_ substrates from Tesla (type KBI2) were used for MoS_2_ flakes deposition. Each sensing substrate includes a Pt 1000 temperature sensor, heater, and platinum interdigitated electrodes structure (IDES) of line/gap width of 15/15 µm. More details on ceramic sensing substrates can be found on the producer’s website (www.tesla-blatna.cz). Solution with MoS_2_ flakes was first subjected to sonication for 30 min to ensure no aggregates. After that, Nordson Precision Fluid Dispenser (type Ultimus Plus II) was used to deposit ten layers of MoS_2_ dispersion onto a cleaned ceramic substrate. After deposition of each layer, the material was dried in airflow (~  50 °C) to evaporate the residuals of ethanol solvent. Ten printing repetitions ensured that the randomly deposited, overlapping MoS_2_ flakes provided the percolation network for electrical measurements. Supplementary Fig. S1 illustrates the deposition process, and Supplementary Table S1 summarizes the printing parameters.

### Optical, structural, electrical, and noise measurements

UV-Vis spectroscopy of the MoS_2_ flakes dispersion was realized using Evolution One Plus UV-Vis two-beam spectrophotometer (Thermo Scientific) with 1-nm wavelength resolution. Optical imaging of the material deposited on glass substrates was performed using a Delta Optical MET-1000-TRF microscope with up to 1000× magnification. For DC measurements, the IDES terminals were connected to the SMUs of the Keithley parameter analyzer (type 4200 A-SCS). Heater terminals were connected with the Keysight E3648A DC power supplier for experiments at an elevated temperature. Gas sensor current–voltage (*I*–*V*) characteristics were measured in the range of 0–20 V. Time-response and low-frequency noise measurements were conducted at 20 V. Noise spectra were collected with a signal analyzer (Stanford Research Systems, model SR785). The signal was amplified (1000×) using a low-noise voltage amplifier (Stanford Research Systems, model SR560). The sensing chamber, batteries, and voltage amplifier were put inside a grounded metal shielding box covered with an amorphous cobalt foil, type MCF5 YSHIELD^®^, to reduce external noise interferences, the effect of ambient light, temperature changes, and laboratory airflow. Due to the high resistance of the MoS_2_ sensor (at least tens of MΩ), voltage fluctuations were measured at the resistor *R* of lower resistance (478 kΩ) connected in series with the sensor. Further, these voltage fluctuations were converted into current fluctuations at the sensor *R*_S_. Monitoring voltage at the resistor *R* enabled the calculation of the current flowing through the resistor *R* and the sensor *R*_S_, which is necessary to normalize the power spectral density of current fluctuations *S*_I_(*f*) for further analysis. We analyzed noise spectra in the frequency range of 0.25–20 Hz collected with a resolution of 0.25 Hz. UV LED (275 nm, type PB2D-1CLA-TC) was positioned close to the sensing surface (less than 0.5 cm) for light-assisted measurements. Setting of the optical power density of the UV LED (0.40–1.59 mW/cm^2^) was obtained by adjusting the polarization current.

### Gas-sensing experiments

The MoS_2_ sensor was placed into the glass chamber for gas-sensing experiments and connected via the Teflon board with the measuring and biasing units to reduce leakage currents. Nitrogen dioxide (NO_2_), ammonia (NH_3_), and acetone (C_3_H_6_O) were used as target gases for the DC characteristics, time response, and low-frequency noise measurements. Each of the selected gases represents a different type of interaction with the semiconducting MoS_2_. NO_2_ is a strongly oxidizing agent, NH_3_ is an electron-donating gas, and acetone is an organic, nucleophilic molecule. Dry synthetic air (S.A.) was used as a carrier gas and a reference atmosphere in our studies. To obtain selected concentrations of target gases, we mixed S.A. with calibrating gases (20 ppm of NO_2_ diluted in N_2_, 30 ppm of NH_3_ diluted in N_2_, or 40 ppm of C_3_H_6_O diluted in N_2_) at specific proportions (e.g., to produce 10 ppm of NO_2_, we mixed 25 mL/min of NO_2_ and 25 mL/min of synthetic air, so as the overall gas flow would be kept at 50 mL/min (50 sccm)). We maintained a constant overall gas flow of 50 mL/min regulated by mass flow controllers, Analyt-MTC, model GFC17, calibrated by the producer to synthetic air as a reference gas. To produce a relative humidity of 40%, dry S.A. was transferred through a glass bubbler with deionized water before reaching the sensing chamber. Sensing experiments were conducted at room temperature (~ 25 °C) or 60 °C and ambient pressure (~ 1 bar).

### Data post-processing and detection limit estimation

The sensor responses were presented as relative changes in the sensor resistance *R*_S_ in reference to the sensor resistance in the carrier gas (S.A.) *R*_0 _via the relation: *S =* (*R*_S_ − *R*_0_)/*R*_0_. The sensor baseline, accompanied by the time drift, was approximated by the exponential function: *S* = *S*_0_ + *ae*^bt^, where *S*_0_, *a*, and *b* are constants, whereas *S* and *t* represent the sensor relative resistive responses and time, respectively, and subtracted from the experimental data. The detection limit (DL) was estimated based on *S* under selected conditions according to the procedure described elsewhere^31^. A third-order polynomial function was used to fit the experimental data points obtained for each target gas. The deviation between experimental and theoretical values of the sensor resistive response was used to estimate the root mean square (RMS). DL was determined using the formula: DL = (S/N)∙RMS/slope, where S/*N* = 3 (signal-to-noise ratio). The slope was derived by fitting the quasi-linear region of each set of data points. Noise spectra were presented as the power spectral density of current fluctuations derived from voltage fluctuations measured at resistor *R* according to the relation: *S*_I_(*f*) = *S*_V_(*f*)/*R*^2^.

## Results and discussion

### Structural and optical characterization of MoS_2_ flakes

**Fig. 1:**
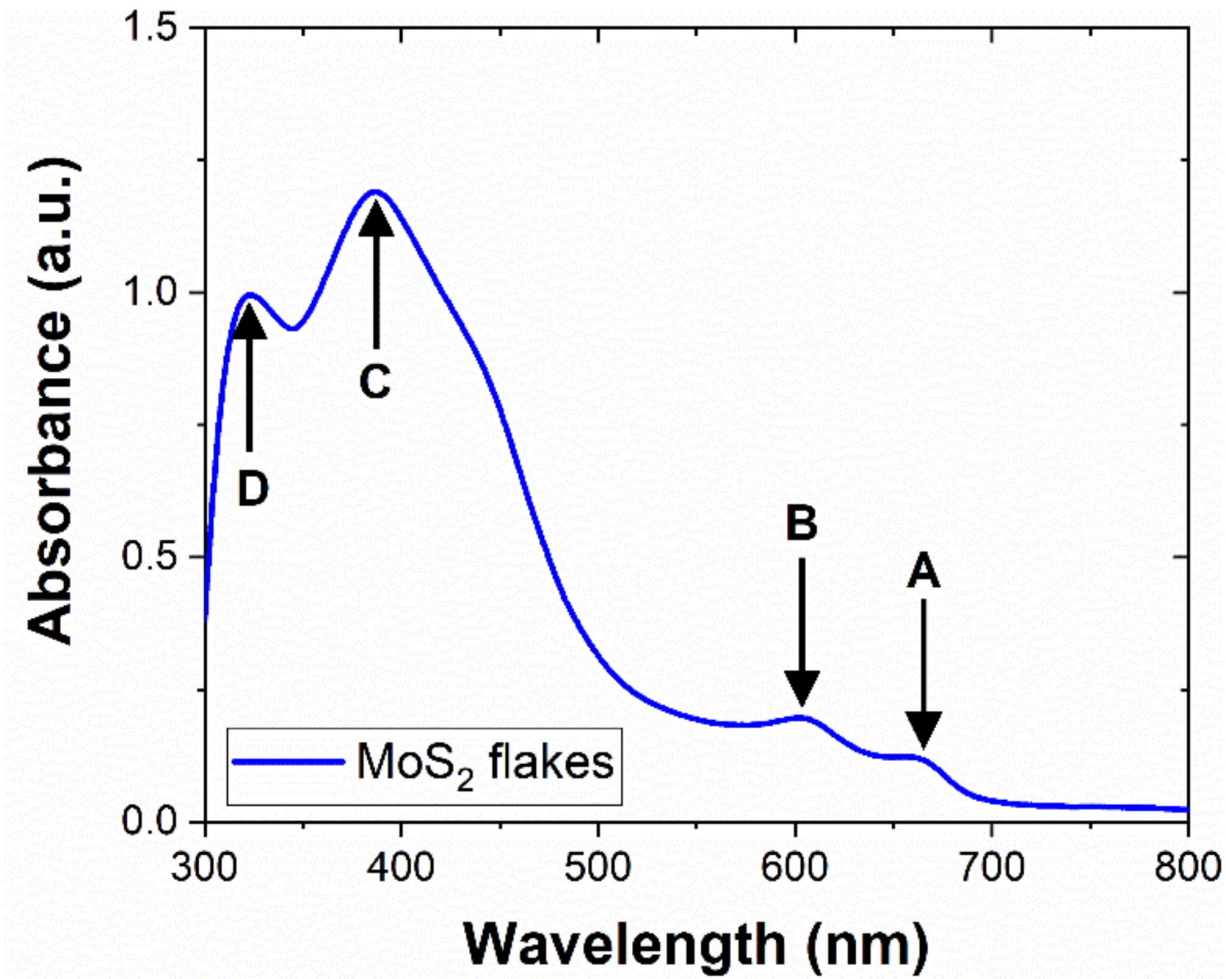
UV-vis absorbance spectrum for MoS_2_ flakes dispersion used for gas-sensitive layers printing. Letters A and B represent optical band transitions independent from the lateral dimensions of the MoS_2_ flakes (660 nm and 602 nm). In contrast, letters C and D represent the excitonic bands ascribed to the nanosized sheets of MoS_2_ (387 nm and 323 nm).

In our work, we study MoS_2_ flakes with a size distribution of 100–400 nm (schematically depicted in Supplementary Fig. S2a). We utilize a ceramic sensing platform that provides temperature control and interdigitated electrodes (IDES) to connect with the printed MoS_2_ layer (see Supplementary Fig. S2b). Figure 1 demonstrates the UV-Vis absorbance of the dispersion after 30 min of sonication to ensure its homogeneity. The absorbance of MoS_2_ flakes was calculated in reference to ethanol solvent. Supplementary transmission spectra for the 300–1000 nm range for MoS_2_ dispersion and ethanol can be found in Supplementary Fig. S3. Four principal local maxima are visible on the absorbance spectrum. Bands A (660 nm) and B (602 nm) represent excitonic transitions in the K point of the Brillouin zone characteristic for a few-layered MoS_2_. The bands designated as C (387 nm) and D (323 nm) correspond to excitonic transitions in the regions of high density of states within the Brillouin zone^32^. The A band at 660 nm corresponds to the energy of ~ 1.9 eV ascribed to the direct optical bandgap in MoS_2_. According to other reports on spectroscopic studies on MoS_2_, the position of the A band can indicate the thickness of the MoS_2_ layers, suggesting that MoS_2_ flakes in the dispersion are mainly bilayers and monolayers in the case of our sensor^33^. While the A and B doublet is associated with the optical properties of the MoS_2_ independent from the lateral size of the structures, the C and D doublet characterizes the nanosized MoS_2_ flakes, and its position can be blue-shifted with the decreasing size of the flakes. Moreover, relatively lower absorbance for the A–B than the C–D doublet can result from the low concentration of MoS_2_ flakes in the solvent.

Transmission optical microscopic images of the MoS_2_ film formed by ten repetitions of printing can be found in Supplementary Fig. S4. Optical imaging confirms the non-uniformity of the deposited structure in the microscale, with thicker parts and aggregates forming due to the simultaneous evaporation of the solvent and the outward flow of the droplet. This results in the coffee ring effect, a common phenomenon for ethanol-based solutions of low viscosity without any additives or binders^27^. More detailed characterization of the MoS_2_ flakes can be found elsewhere^34^.

### DC resistance measurements

**Fig. 2:**
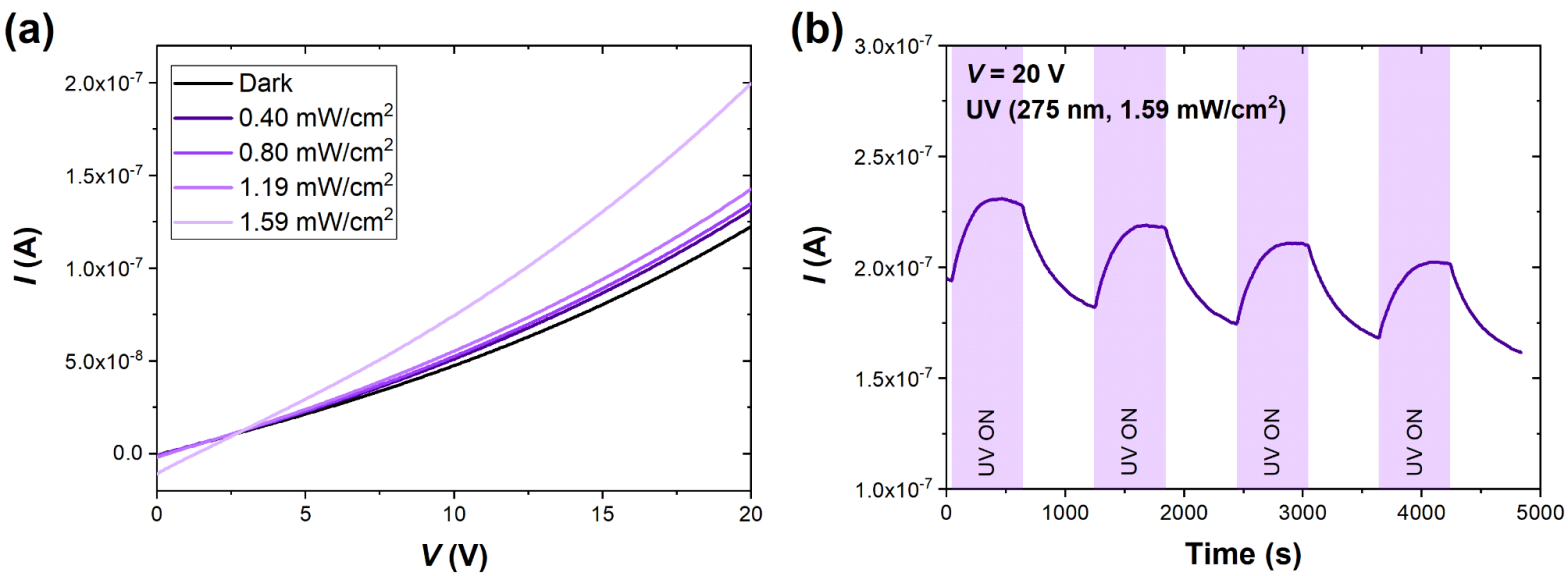
(**a**) Current–voltage (*I*–*V*) characteristics of MoS_2_ sensor in S.A. for different optical power densities of the UV LED (275 nm), and (**b**) time response (current) of MoS_2_ sensor to four consecutive cycles of UV light (275 nm, 1.59 mW/cm^2^) irradiation. Each irradiation cycle lasted 10 min, followed by 10 min of recovery in the dark. The bias voltage was set to 20 V for time-response measurements.

Since UV light has been repeatedly reported to influence the conductance of the MoS_2_ monolayers^35^, we opted for a UV-C LED (maximum optical power at 275 nm) for light-assisted measurements. We started the electrical measurements by studying the effect of the optical power density of the UV light on the MoS_2_ electrical characteristics. Figure 2a shows the current-voltage characteristics (*I*–*V*) of the MoS_2_ sensor in synthetic air (S.A.) under UV irradiation of optical power density between 0.40 and 1.59 mW/cm^2^. We kept the constant distance between the UV LED and the sensing surface (~ 0.5 cm) for these measurements. The maximum optical power density investigated was 1.59 mW/cm^2^, which provided the most significant effect on the current flowing through the sensor and was selected for further UV-assisted experiments with target gases. Moreover, the *I*–*V* curve shows a deviation from the ohmic behavior in the 0–20 V range, which is expected for the structure consisting of multiple semiconducting nanoflakes overlapping each other and creating a percolation network. Figure 2b demonstrates the current response to four consecutive cycles of UV irradiation (275 nm, 1.59 mW/cm^2^) ON/OFF switching. We observed the constant time drift towards lower currents, but the relative change in the current (peak-to-peak values) due to irradiation was the same, i.e., between 18 and 20% in each cycle. The values were derived as (*I*_ON_–*I*_OFF_)/*I*_OFF_, where *I*_ON_ is the current flowing through the irradiated sensor (the last point from the considered irradiation cycle), and *I*_OFF_ is the current flowing through the dark sensor (the last point from the cycle in the dark)—see how the current responses were derived from Fig. 2 in Supplementary Material (Supplementary Fig. S5). Similar results were reported by Kumar et al. for the MoS_2_ sensor, which exhibited ~ 18% stable response under UV irradiation^17^. The reason for the observed drift can be that printed MoS_2_ is not a single atomic layer but is formed by multiple nanoflakes. Thus, the resistance of individual flakes and the structure boundaries contribute to the overall sensor resistance. Applying higher voltage and inducing photocurrent in the structure might cause the elongated stabilization process, mainly when UV light activates surface cleaning by removing preadsorbed oxygen and humidity. Therefore, ink-printed MoS_2_ layers may exhibit longer photocurrent relaxation and less pronounced saturation in the air compared to individual atomically thin flakes^36^.

**Fig. 3:**
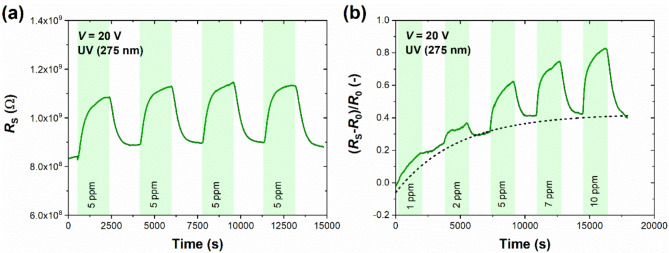
(**a**) Time response (resistance) of MoS_2_ sensor to four consecutive cycles of NO_2_ (5 ppm) introduction; and (**b**) time response (relative changes in resistance) of MoS_2_ sensor to five cycles of NO_2_ of selected concentrations (1–10 ppm) under UV light (275 nm). Each detection cycle consisted of 30 min-response and 30 min-recovery in S.A. The bias voltage was set to 20 V. The black dashed exponential curve in (**b**) denotes the time drift present during detection.

Before gas sensing experiments, we cleaned the sensor surface for approximately one hour under UV irradiation in S.A. to reduce the short-time drift and stabilize the baseline to some extent. We started gas sensing experiments with nitrogen dioxide (NO_2_), representing the inorganic oxidizing species harmful to the environment and humans even at the ppb-concentration level^37^. Although NO_2_ usually bonds strongly with the sensing surfaces of nanomaterials via physical forces, room-temperature sensing usually requires an additional boost to maximize the sensitivity toward the gas and minimize the detection limit. UV light successfully fulfilled these requirements for graphene and MoS_2_ sensors^38–40^. Figure 3 summarizes the qualitative and quantitative detection of NO_2_ by printed MoS_2_ sensor. Consecutive cycles of introducing 5 ppm NO_2_ resulted in repeatable responses (the average peak-to-peak resistive response of ~ 28%) with time drift reduced after the first detection cycle (Fig. 3a). For quantitative detection, the sensor was subjected to NO_2_ concentrations between 1 and 10 ppm. Figure 3b presents the relative changes in the sensor resistance *R*_S_ in reference to resistance in S.A. NO_2_ increases the sensor resistance, which agrees with the oxidizing nature of NO_2_ interacting with the *n*-type semiconductor. When NO_2_ molecules adsorb on the MoS_2_ surface, they extract electrons and reduce the number of majority carriers in the sensing material, resulting in higher resistance. UV excitation provides additional light-generated electrons that may participate in molecular detection but also increases the probability of adsorption by cleaning the binding sites occupied by the oxygen and humidity species from the atmospheric air. It can also be noticed that sensing smaller concentrations of NO_2_ (1 and 2 ppm) is subjected to more intense time drift. We observed that the sensor responded reproducibly when the experiment was repeated in the following days after staying in laboratory air during the night, necessary for its full recovery. Similar drift components were observed elsewhere^41^. As the baseline drift of the MoS_2_ sensor was observed to diminish in time, suggesting the process with a particular relaxation time, it was fitted to the exponential function described in Methods. The processed data represents the actual responses to the target gas without the influence of the drifting baseline. The results of the data processing for NO_2_ are depicted in Supplementary Fig. S6. Another ink-printed MoS_2_ sensor was also tested toward NO_2_ (5 ppm) in the dark and under UV light (see Supplementary Fig. S7). Resistive response in the dark was only 4% but reached ~ 20% under UV light for 15-min exposure to NO_2_ (at *V* = 20 V). This observation confirmed the superiority of UV-assisted sensing; therefore, we continued with UV light sensing only in all following experiments.

**Fig. 4:**
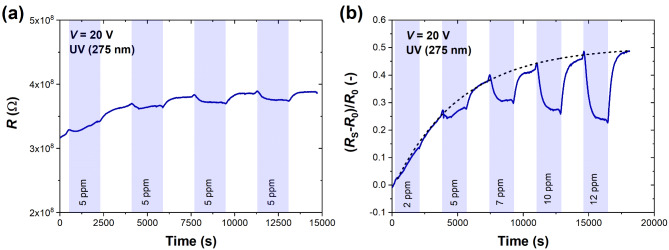
(**a**) Time response (resistance) of MoS_2_ sensor to four consecutive cycles of NH_3_ (5 ppm) introduction; and (**b**) time response (relative changes in resistance) of MoS_2_ sensor to five cycles of NH_3_ of selected concentrations (2–12 ppm) under UV light (275 nm). Each detection cycle consisted of 30 min-response and 30 min-recovery in S.A. The bias voltage was set to 20 V. The black dashed exponential curve in (**b**) denotes the time drift present during detection.

Figure 4 shows the effect of ammonia (NH_3_) on the studied device. The repeatable resistance decrease toward 5 ppm of NH_3_ can be visible in Fig. 4a. Ammonia is a reducing gas with a lone-pair electron acting as an electron donor to MoS_2_. Similarly to the NO_2_ case, a substantial time drift, particularly in low concentrations of NH_3_ (2 and 5 ppm), is visible in Fig. 4b. The stabilized sensing response is visible for 7 ppm and higher concentrations, where the sensor responsivity is much stronger. The resistive responses to 2–12 ppm NH_3_ after data processing and subtracting the drifting baseline are depicted in Supplementary Fig. S8. Here, the decreasing resistance toward subsequent cycles of NH_3_ detection is much more apparent and simplifies the interpretation of the direction of changes in MoS_2_ electrical properties after the adsorption of the reducing gas.

**Fig. 5:**
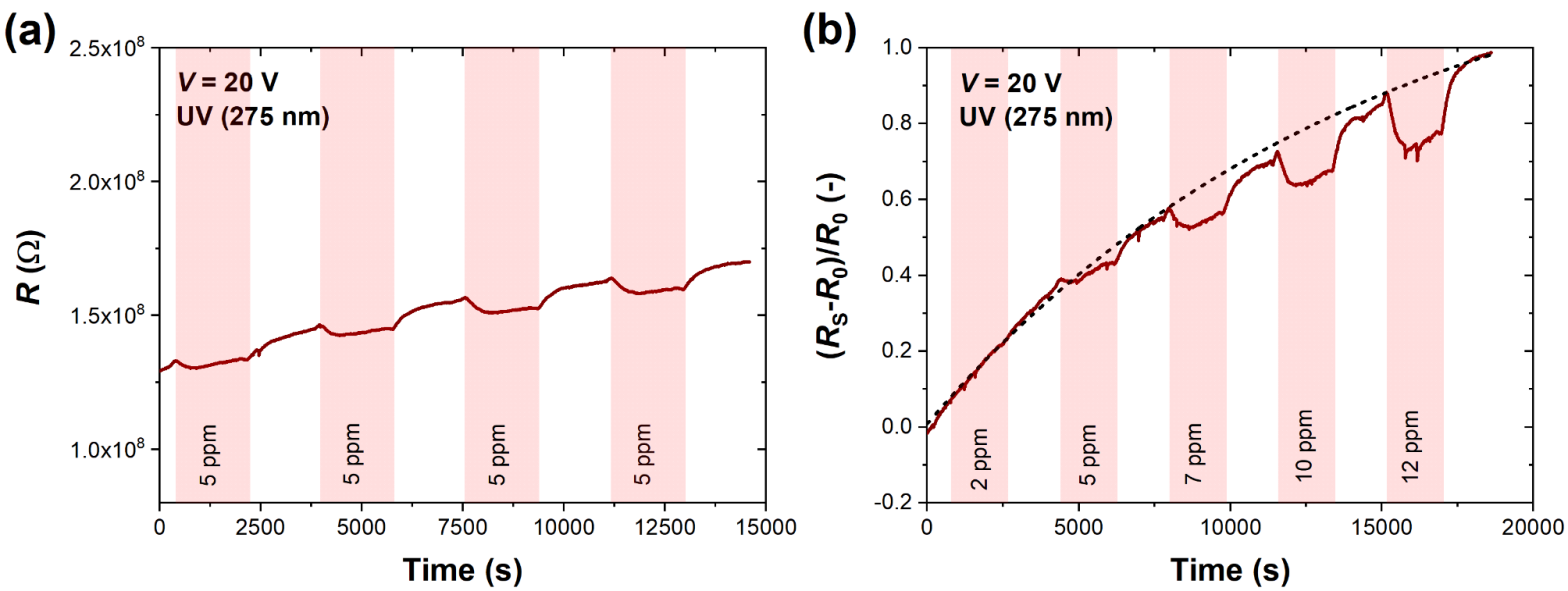
(**a**) Time response (resistance) of MoS_2_ sensor to four consecutive cycles of C_3_H_6_O (5 ppm) introduction; and (**b**) time response (relative changes in resistance) of MoS_2_ sensor to five cycles of C_3_H_6_O of selected concentrations (2–12 ppm) under UV light (275 nm). Each detection cycle consisted of 30 min-response and 30 min-recovery in S.A. The bias voltage was set to 20 V. The black dashed exponential curve in (**b**) denotes the time drift present during detection.

A similar reduction of the sensor resistance was observed during acetone (C_3_H_6_O) detection. Usually, organic vapors bind weakly with the surfaces of low-dimensional materials and exhibit relatively low adsorption energies^42,43^. Figure 5a shows repeatable cycles of 5 ppm acetone introduction under continuous UV irradiation, and Fig. 5b demonstrates the sensor responsivity toward increasing acetone concentration (2–12 ppm). Both measurements are accompanied by time drift, which is more pronounced than for NO_2_ and NH_3_ cases. After fitting and subtracting the drifting baseline (see Supplementary Fig. S9), one can notice that acetone exhibits reducing properties during interaction with the MoS_2_ surface, similar to ammonia. Comparing the same concentration range for both reducing gases, we can observe that inorganic ammonia produces higher and more stable responses in each detection cycle than organic acetone. Nevertheless, the resistive responses reach up to ~ 20% for 12 ppm of acetone, and the obtained results are higher than reported before for pure MoS_2_ under UV light at RT^44^. Based on the DC response studies, we can see that the sensor responsivity is superior to oxidizing NO_2_ rather than to reducing gases (see Supplementary Fig. S10 illustrating the sensor selectivity based on the time-resolved resistance monitoring). In terms of response and recovery times, there is no strict correlation with the type or concentration of gas, as shown in Supplementary Fig. S11, and time constants are at least in the several-minute range. Additionally, introducing relative humidity (RH) of 40% during sensing of 5 ppm of NO_2_, NH_3_, and C_3_H_6_O showed that resistive responses were slightly increased in a humid atmosphere. However, in dry S.A., the sensor maintained its responsivity toward selected target gases after four months of storage in laboratory air conditions. The results showing the sensor stability and the effect of RH = 40% are presented in Supplementary Table S2 and Supplementary Fig. S12.

**Fig. 6:**
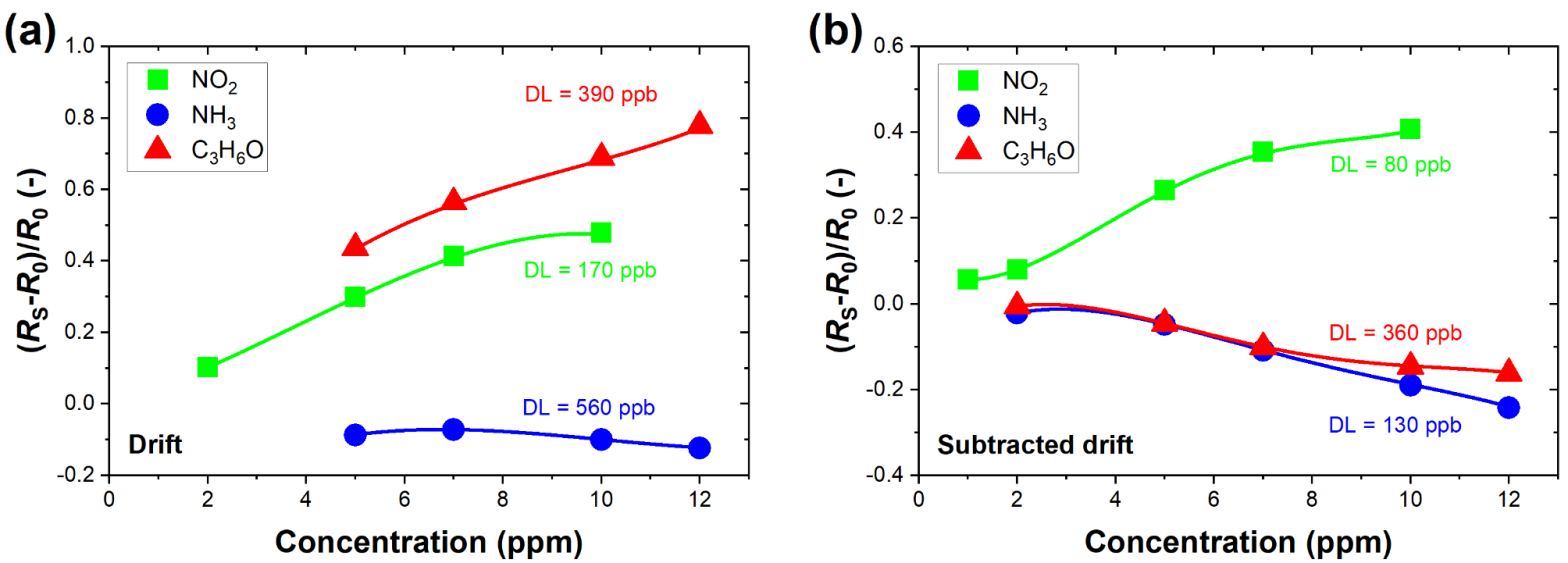
Relative changes in MoS_2_ sensor resistance as a function of target gas concentration for NO_2_, NH_3_, and C_3_H_6_O under UV light (275 nm) (**a**) before and (**b**) after subtraction of the drift baseline. Theoretical detection limits established for NO_2_, NH_3_, and C_3_H_6_O are marked on the graphs and are equal to 170 ppb, 560 ppb, 390 ppb (drift case), and 80 ppb, 130 ppb, and 360 ppb (subtracted drift case), respectively. Solid curves correspond to a third-order polynomial function fitted to the experimental points for DL estimation. The responses to acetone burden with drift do not show the real direction of changes in sensor resistance.

Another crucial figure of merit for a gas sensor is the detection limit (DL), which informs about the maximum sensitivity to a specific target gas. Here, DL was estimated before and after subtracting the baseline drift to see if post-processing of detection data improves DL for the MoS_2_ sensor. Figure 6 compares DL values for NO_2_, NH_3_, and C_3_H_6_O calculated based on relative resistive responses (*R*_S_ − *R*_0_)/*R*_0_ according to the procedure described in Methods. The authors designated the pre-processed and post-processed data points as the “drift” and “subtracted drift” cases. For the drift case, the response data measured for the lowest concentration of each gas (1 ppm for NO_2_ and 2 ppm for NH_3_ and C_3_H_6_O) was purposely omitted as these points were the least readable out of the drifting baseline, making the DL analysis much less reliable. Indeed, the time drift influences the sensing responses. If the target gas increases the sensor resistance (oxidizing NO_2_), the post-processed data points reveal slightly lower responses. On the other hand, if the gases decrease the sensor resistance (reducing NH_3_ and acetone), their responses become more pronounced and readable, constituting a much more reliable representation of the actual adsorption process and direction of charge transfer occurring between MoS_2_ flakes and target gases. Despite the drifting baseline, Fig. 6a demonstrates that all three gases exhibit sub-ppm DL, with the lowest value of 170 ppb obtained for NO_2_. However, due to the substantial time drift and weakly binding gas molecules, the DL values calculated for reducing gases are burdened with a significant error, particularly for acetone. Hence, the “subtracted drift” case presents more reliable results (Fig. 6b). DL values calculated after data processing are 80 ppb, 130 ppb, and 360 ppb for NO_2_, NH_3_, and C_3_H_6_O, respectively. This means that for NO_2_ and NH_3_, the DL was improved more than 2 and 4 times, respectively, only by simple data post-processing. This suggests an easy method of improving the performance of sensors based on solution-processed, ink-printed MoS_2_ flakes. The DL estimated for NO_2_ and NH_3_ falls significantly beyond the maximum permissible limits set by the European Union, which are 0.5 ppm and 1 ppm for 8-h and 15-min exposure for NO_2_, and 20 ppm and 50 ppm for the long and short exposure for NH_3_^45^. Up to the authors’ knowledge, in the case of acetone, the sub-ppm limit was reached only at elevated temperatures (0.1 ppm at 300 °C–100 °C with additional UV irradiation), and it was reported for HZnO/MoS_2_ hybrid sensors^44^. For pure MoS_2_, the DL for acetone reaching lower than 0.9 ppm still requires further investigation^1^, making our proposed sensor promising for the efficient detection of this organic gas.

To fulfill the investigation on possible methods of improvement of sensitivity for printed MoS_2_ sensors, measurements at an elevated temperature (60 °C) were performed for all three target gases. Interestingly, the elevated temperature did not improve the MoS_2_ sensor performance. On the contrary, only an unstable drift upwards was observed for 5 ppm of NO_2_, NH_3_, and acetone (see Supplementary Fig. S13 for time responses under 60 °C and UV irradiation and proposed explanation for this observation).

### Low-frequency noise measurements

**Fig. 7:**
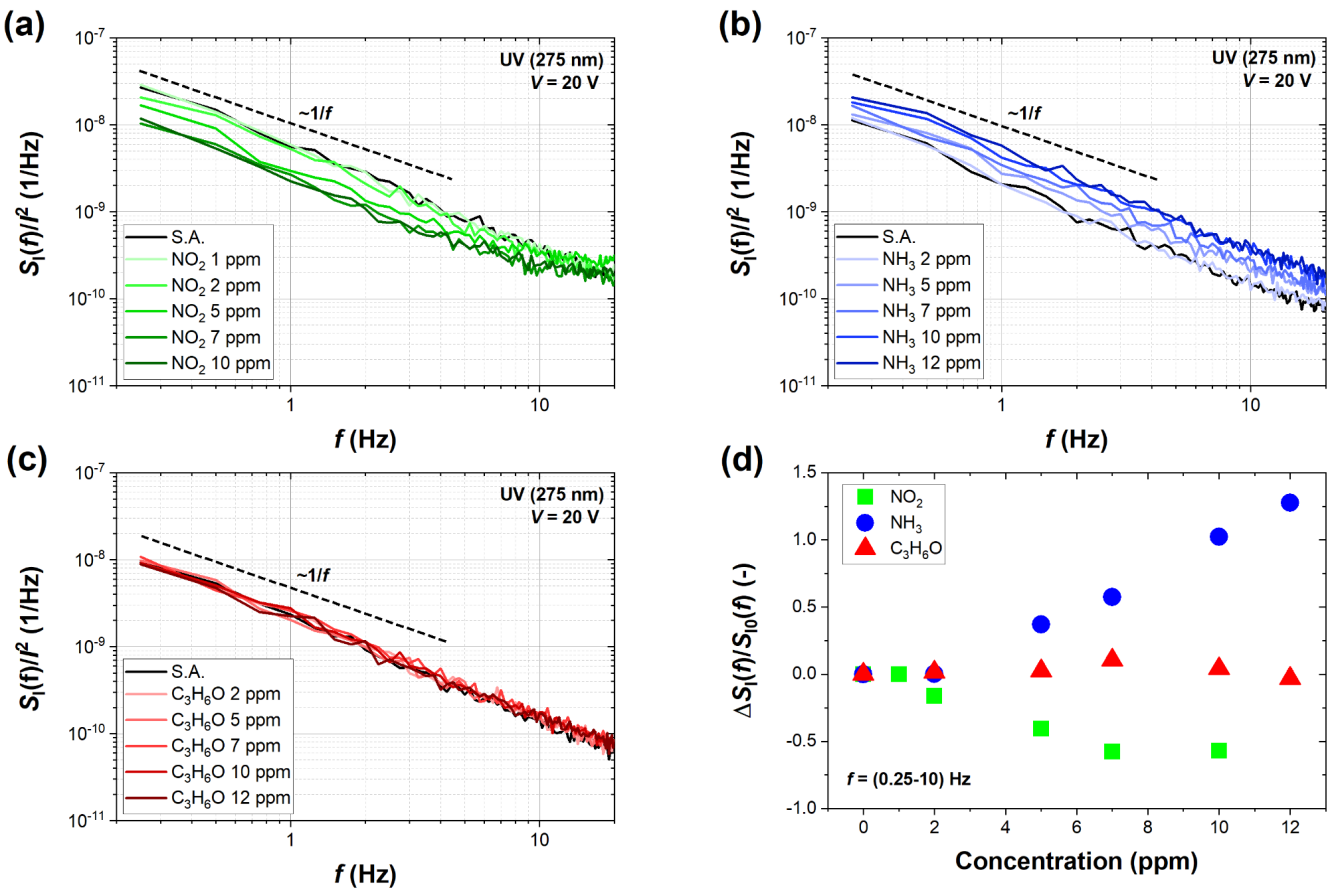
Power spectral density of current fluctuations *S*_I_(*f*) normalized to the squared bias current *I*^2^ for selected concentrations of (**a**) NO_2_, (**b**) NH_3_, and (**c**) C_3_H_6_O. The dashed lines correspond to the 1/*f* noise dependence. The spectra were measured under UV light (275 nm) at voltage bias *V* = 20 V across the sensor. Data points in (**d**) correspond to the relative changes Δ*S*_I_(*f*)/*S*_I0_(*f*) in the noise current power spectral density in the target gas of selected concentration Δ*S*_I_(*f*) = *S*_I_(*f*)-*S*_I0_(*f*) in relation to the reference case of S.A. *S*_I0_(*f*) (the values are averaged for frequencies 0.25–10 Hz).

Fluctuation-enhanced sensing was another method to investigate the sensing performance of the UV-assisted MoS_2_ sensor. Low-frequency noise was measured and analyzed up to 20 Hz, where the 1/*f* noise was observed to be the dominant noise component over background and white noise. Noise spectra were collected for different concentrations of NO_2_, NH_3_, and C_3_H_6_O that corresponded to the DC measurements and are depicted in Fig. 7. The adsorption of oxidizing NO_2_ decreases the normalized current noise power spectrum *S*_I_(*f*)/*I*^2^ with the gas concentration increase. Such direction of sensor responses correlates with the decrease in the current flowing through the sensor caused by NO_2_. The opposite effect can be observed for ammonia since higher concentrations of NH_3_ visibly increase the current fluctuations. In the case of the organic acetone, the noise responses to this gas are minor, even for the highest concentrations investigated. The results indicate that the adsorption of only some gases of the considered low concentrations induces distinct fluctuations, and the FES method can be used for determining the concentration of NO_2_ and NH_3_. However, the range of concentrations can be limited and differ for various gases, e.g., 1 ppm of NO_2_ and 2 ppm of NH_3_ produce only minor changes in the noise intensity. Additionally, Fig. 7d demonstrates the magnitude of the relative changes introduced in the noise of the MoS_2_ sensor. Based on these results, it is easy to distinguish between oxidizing and reducing gas (NO_2_ and NH_3_) due to the opposite direction of responses. The noise response to NO_2_ saturates from 7 ppm, reaching around − 0.6. On the contrary, noise amplitude increases proportionally, with NH_3_ concentration reaching ~ 1.3, showing a high linearity of sensor responsivity. The saturation for NH_3_ is likely to be achieved for much higher concentrations, whereas in the case of NO_2_, FES can be utilized efficiently toward the range of lower concentrations (below 7 ppm). In the case of acetone, no relevant changes in the noise intensity suggest that such low concentrations of this gas cannot be detected with sufficient accuracy based on the 1/*f* noise spectra. Interestingly, the sensitivity of noise spectra to higher concentrations of acetone (110 ppm) and other organic vapors was reported recently for the ink-printed MoS_2_, suggesting that organic species can produce distinct noise spectra but at much higher concentrations^18^. It can be explained by their low binding energies (adsorption energies) to different types of materials and limited charge transfer, which is the primary mechanism of gas detection by 2D semiconducting metal sulfides. Moreover, noise responses for NO_2_ and NH_3_ are higher than DC resistance responses with subtracted drift baseline. For instance, 10 ppm of NO_2_ induces the absolute value of resistive response of ~ 0.4 and noise response of ~ 0.6, an increase of 50%. For 12 ppm of NH_3_, this increase is even more apparent as the noise response is more than six times higher (~ 1.3) than the resistive response (~ 0.2). At the same time, the adsorption of NO_2_ and NH_3_ does not induce any characteristic events in the form of Lorentzians dominant over the 1/*f* noise spectrum, as was observed for graphene-based sensors^20^. Moreover, it was previously confirmed that low-frequency noise spectra of monolayers and bilayers of MoS_2_ respond poorly to selected organic vapors (e.g., ethanol, methanol, toluene), and the increased noise intensity was observed only to acetonitrile^46^. However, no quantitative sensing has been reported for this material, specifically for ink-printed light-assisted MoS_2_. This makes room for original research and discoveries, and our results pave the way toward novel applications for this type of materials and the FES method.

## Conclusions

MoS_2_ sensors were fabricated employing the ink-printing of nanosized flakes dispersed in an ethanol-water solution. The optical characterization of the dispersion revealed that it primarily consists of bilayered and monolayered MoS_2_ with direct optical bandgap in the red-light region of the spectrum (660 nm). The optical imaging confirmed that the printed structure is formed with sub-µm flakes, non-uniformly deposited on the ceramic substrate. Despite the coffee-ring effect, the size of the deposited droplets larger than the IDES area provided a uniform distribution of flakes and reduced aggregation of the material (compared to the edges of the droplet) in the region of interest for electrical and low-frequency noise measurements. Printing repetitions and deposition of the 10-layered structure ensured the percolation path between nanoflakes printed onto a ceramic platform with platinum electrodes of 15 μm-gap for electrical measurements. The deposited MoS_2_ structure exhibited ~ 10^8^ Ω resistance at the biasing voltage of 20 V and under UV irradiation (275 nm, 1.59 mW/cm^2^). The UV light ensured stable current flow through the sensor; however, it did not completely diminish the short-time drift of the sensor resistance. The drifting baseline specifically affected the detection of reducing gases (ammonia and acetone) and disturbed the interpretation of the direction of changes introduced by these species. Data post-processing and subtraction of the drifting baseline enabled the reduction of the detection limit to 80 ppb, 130 ppb, and 360 ppb for NO_2_, NH_3_, and C_3_H_6_O, respectively, showing the possibility of enhancing sensitivity for printed MoS_2_ gas sensors at RT. In compliance with the redox properties of target gases, NO_2_ increased the sensor resistance, whereas NH_3_ and acetone reduced it, confirming the *n*-type conductivity of the MoS_2_ structure. Low-frequency noise measurements reveal that FES can be utilized to determine the concentration of NO_2_ and NH_3_ for the linear range of the changes induced in the noise amplitude (below 7 ppm for NO_2_ and up to 12 ppm of NH_3_). Supplementary Table S3 summarizes the properties of MoS_2_-based gas sensors operating at RT and utilizing UV light assistance reported recently, showing that ink-printed MoS_2_ sensor offers simplicity of its fabrication while still preserving the high sensitivity and selectivity increased with the FES method. Combining DC resistance measurements with the FES has the potential to become a highly sensitive tool for light-assisted gas detection by ink-printed MoS_2_ and similar structures.

## Electronic supplementary material

Below is the link to the electronic supplementary material.

Supplementary Material 1

## Data Availability

The detailed data that support the findings of our experimental study are available from the corresponding author upon reasonable request.
