# Supplementary material for: UV-assisted fluctuation-enhanced gas sensing by ink-printed MoS2 devices
Source: Sci Rep. 2024 Sep 27;14:22172. doi: 10.1038/s41598-024-73525-2 (PMC11437144; doi:10.1038/s41598-024-73525-2)
Supplement: Supplementary file 1 — Supplementary Material 1 [file 41598_2024_73525_MOESM1_ESM.docx]

***Supplementary Material***

**UV-assisted fluctuation-enhanced gas sensing by ink-printed MoS_2_ devices**

Katarzyna Drozdowska^a,*^, Janusz Smulko^a^, Jakub Czubek^a^,
Sergey Rumyantsev^b^, Andrzej Kwiatkowski^a^

*^a^Department of Metrology and Optoelectronics, Faculty of Electronics, Telecommunications, and Informatics, Gdańsk University of Technology, G. Narutowicza 11/12, 80-233, Gdańsk, Poland*

*^b^CENTERA Laboratories, Institute of High Pressure Physics PAS, Warsaw, Poland*

*Corresponding author – katarzyna.drozdowska@pg.edu.pl

Figure S1 illustrates the fabrication of MoS_2_ sensing layers *via* printing from the MoS_2_ flakes dispersion onto ceramic substrates (Figure S1a) and liquid flow and solvent evaporation responsible for the morphology of the obtained printed layers. Low-viscosity inks based on ethanol-water solutions tend to form a coffee ring on the substrate, resulting in films thicker at the edges than in the center of the droplet. Table S1 summarizes the printing parameters for the deposition of MoS_2_ films.

**
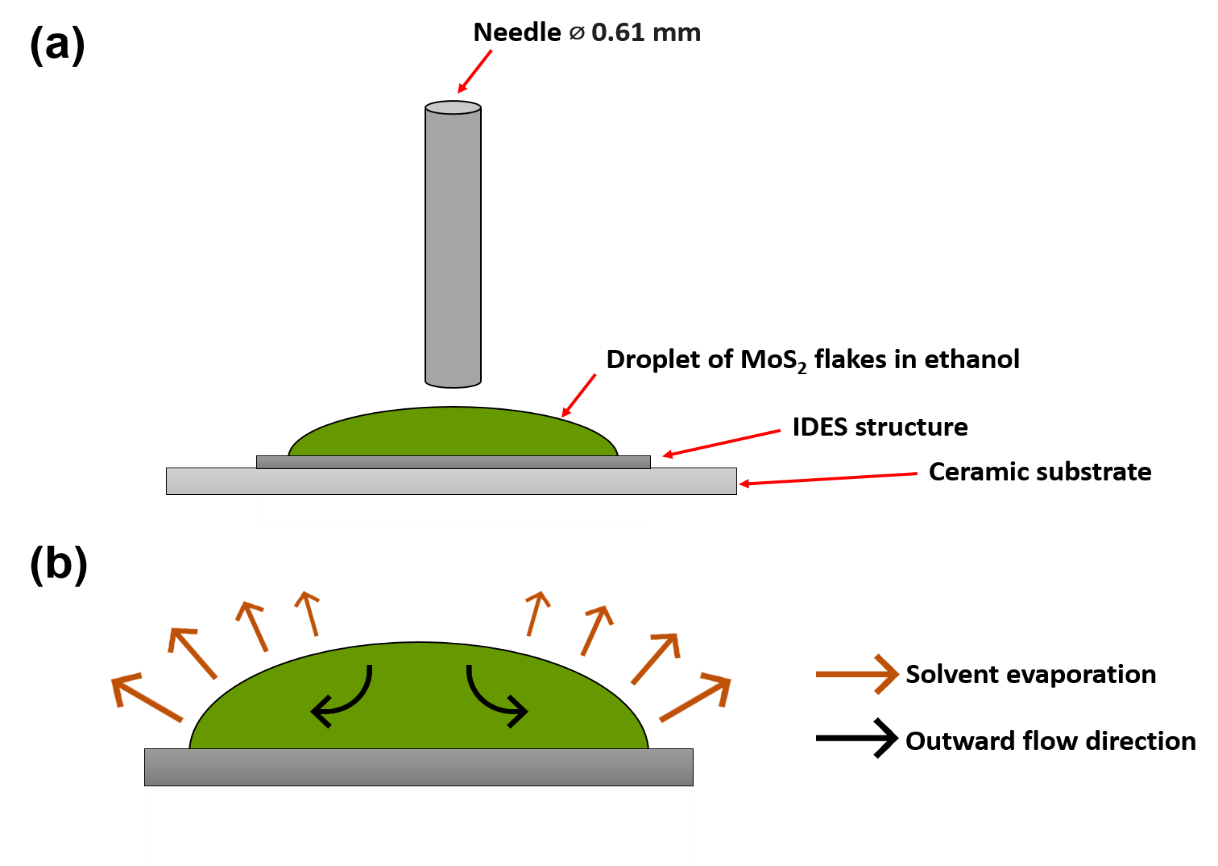
**

**Figure S1** Schematic representation of the MoS_2_ layers printing procedure: **(a)** MoS_2_ dispersion deposited onto a ceramic substrate with interdigitated electrodes (IDES) using the needle of the 0.61 mm inner diameter; **(b)** the fastest solvent evaporation and accelerated aggregation of MoS_2_ at the edges of the droplet results in the coffee ring effect of non-uniform flakes distribution.

**Table S1** Printing parameters for MoS_2_-based sensors.

| Temperature of the substrate/dispersion | RT/RT |
| --- | --- |
| Drying temperature | ~50 °C |
| Carrier gas pressure | 0.6 psi (~0.04 bar) |
| Time of deposition (releasing a single droplet) | 0.05 s |

Figure S2a depicts MoS_2_ flakes deposited randomly on the substrate with a size distribution of 100–400 nm, according to the producer. The S-Mo-S plane depicted in Figure S2a resembles the honeycomb lattice of graphene, and similarly, it is defined by covalent intralayer bonds that form the stable 2D structure. Figure S2b depicts the schematic view of the ceramic sensing platform that provides temperature control and interdigitated electrodes (IDES) to connect with the printed MoS_2_ layer. The UV LED (275 nm) close to the sensing surface facilitates light-assisted detection.

**
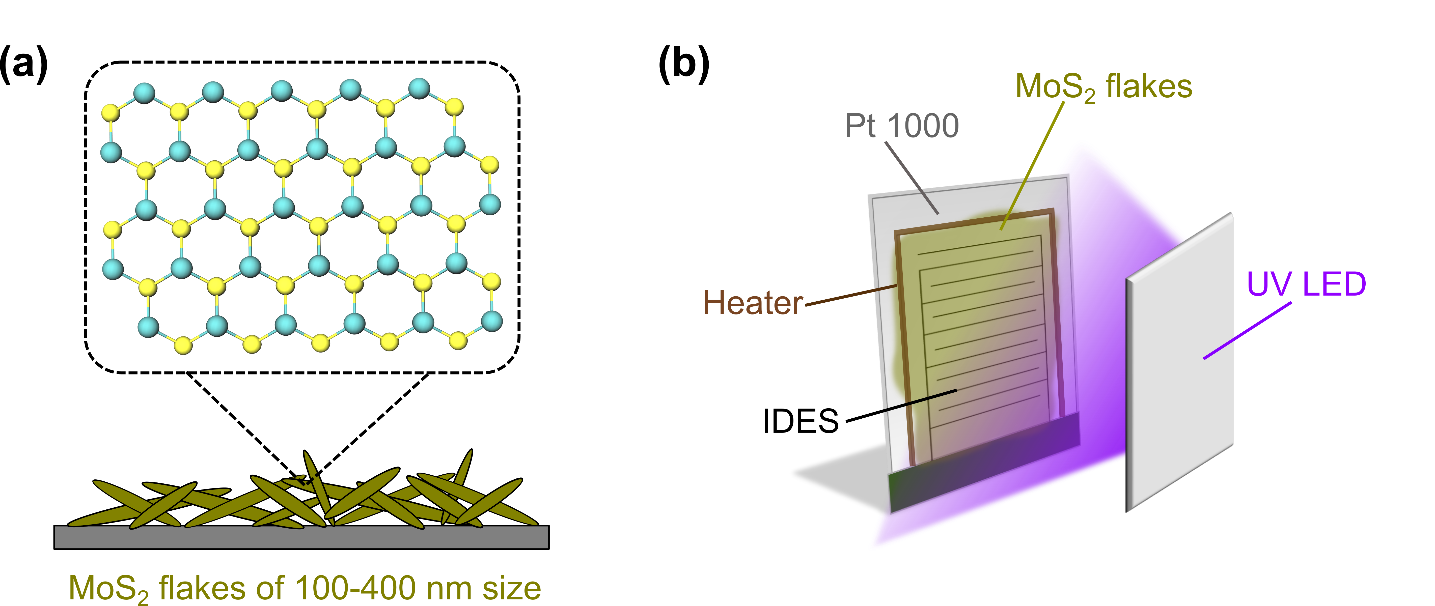
**

**
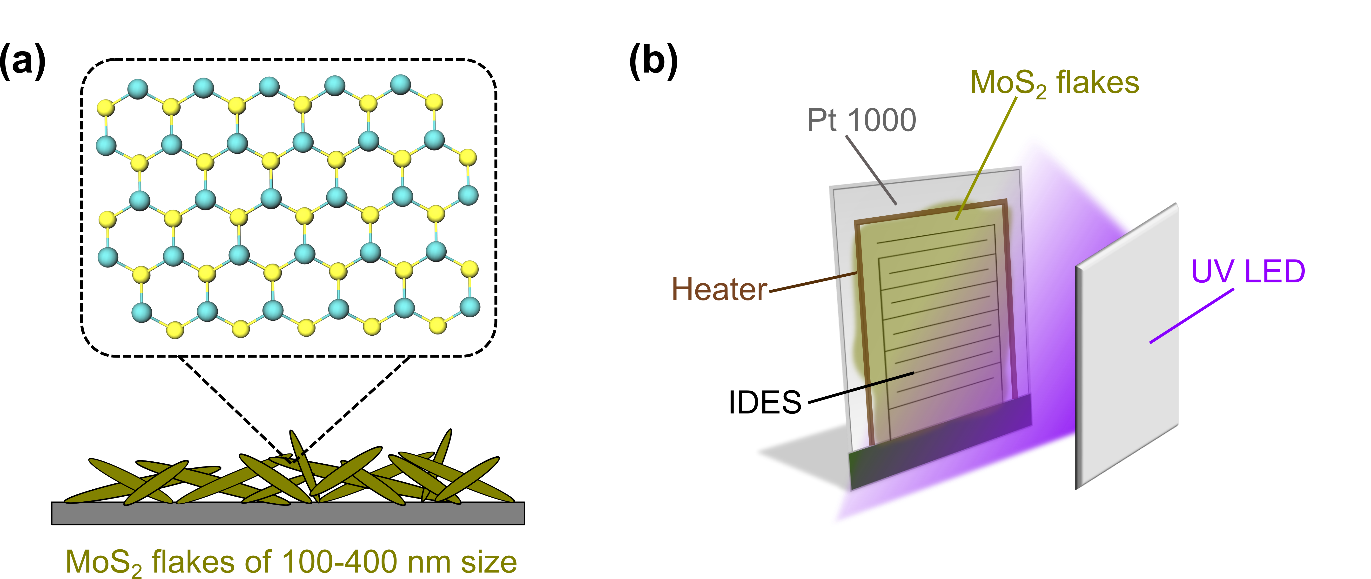
**

**Figure S2 (a)** The depiction of MoS_2_ flakes deposited on a ceramic substrate showing
a randomly oriented structure with a top view of the 2D MoS_2_ monolayer with S-Mo-S atomic structure (yellow atoms represent sulfur and cyan atoms represent molybdenum); and **(b)**
a schematic view of the sensing platform with MoS_2_ flakes deposited onto IDES and irradiated with UV light.

Figure S3 shows the transmittance spectrum for MoS_2_ flakes dispersion used for gas-sensitive layers fabrication and ethanol as the reference spectrum. The visible light range is dominated by the absorption of MoS_2_ flakes since the ethanol spectrum is characterized by an almost constant transmittance in this range. The bands identified on the MoS_2_ spectrum in the visible light range relate to the excitonic transitions ascribed to optical bandgap transitions (600–700 nm) or dependent on the structural properties (lateral size) of the nanoflakes (300–400 nm). The local transmittance minima in the near-infrared part of the spectrum are associated with the absorption and scattering by O-H bonds from the solvent, whereas the UV range is dominated by the absorption of the quartz cuvette used for the measurements – the absorption edge of quartz is visible particularly in the ethanol spectrum as an abrupt drop of transmittance at ~300 nm. Based on the transmittance (in %) of the dispersion *T*_D_ and the solvent *T*_S_, the absorbance *A* of MoS_2_ flakes in reference to the solvent can be derived as the difference in absorbance of the whole dispersion *A*_D_ and the solvent itself *A*_S_ from:

*A* = *A*_D_ – *A*_S_ = log(100/*T*_D_) – log(100/*T*_S_).





**Figure S3** UV-vis transmittance spectra in 200–1000 nm range for ethanol and MoS_2_ flakes dispersion in ethanol-water solution. The visible light range is dominated by the MoS_2_ absorption connected with the optical and structural properties of the 2D flakes.

Figure S4 shows two images from a transmission optical microscope of a 10-layered MoS_2_ nanoflakes structure fabricated *via* ink-printing with a precise liquid dispenser in the middle (top image) and at the edge (bottom image) of the formed droplet. The images indicate that the fabricated layer consists of sub-micrometer flakes that tend to accumulate non-homogeneously on the substrate, particularly on the edges (coffee-ring effect).

**
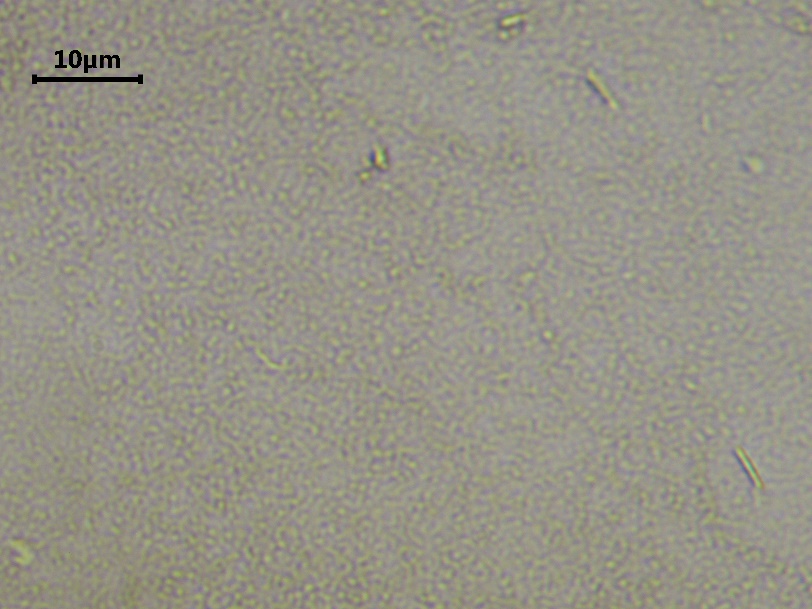
**

**
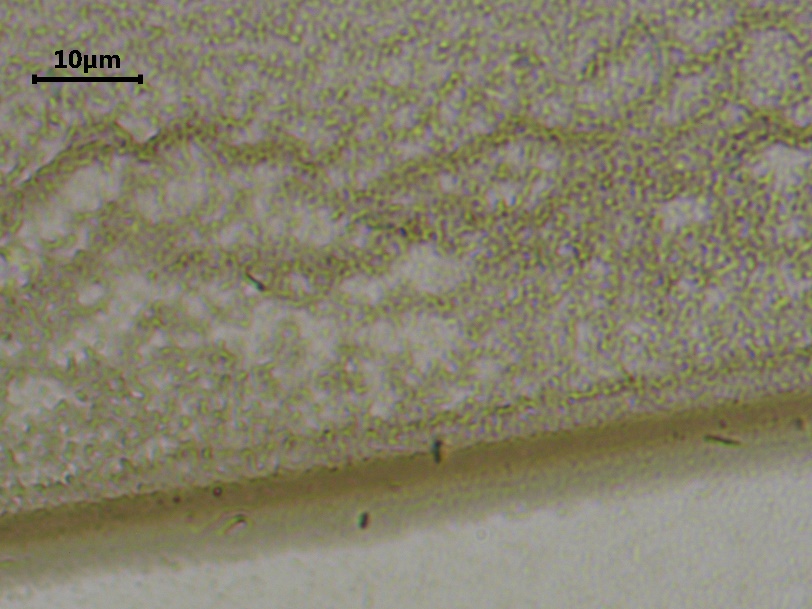
**

**Figure S4** Transmission optical microscope images (1000x magnification) of 10-layered MoS_2_ structure consisting of nanoflakes (average size between 100–400 nm according to the producer – Graphene Supermarket). A 10-layered MoS_2_ structure was deposited onto a glass substrate for microscopic imaging.

Figure S5 depicts how the current response of the irradiated sensor was measured in four consecutive cycles of UV light ON/OFF switching. The values in % are current responses measured based on the last data point from the irradiated cycle (*I*_ON_) and dark cycle (*I*_OFF_). The calculated values for the observed time range are between 17.5% and 20.7%.





**Figure S5** Time response (current) of MoS_2_ sensor to four cycles of UV light ON/OFF switching (275 nm, 1.59 mW/cm^2^). Each detection cycle consisted of 10 min-response (ON cycle) and 10 min-recovery (OFF cycle) in S.A. The bias voltage was set to 20 V. Red dots are markers for the *I*_ON_ and *I*_OFF_ data points used to calculate current response according to the formula presented in the figure for each irradiation cycle.

Figure S6 presents the MoS_2_ sensor time response to selected concentrations of NO_2_ under UV irradiation (275 nm) after subtraction of the baseline drift. The apparent increase in the sensor resistance confirms the *n*-type conductivity of MoS_2_ flakes. In general, NO_2_ is
a highly electrophilic molecule, accepting electrons from the MoS_2_, which results in a resistance increase:

|  | $NO_{2 \left( \mathrm{gas} \right)}+ e^{-}\to{NO}_{2 (ads)}^{-}.$ | (1) |
| --- | --- | --- |

Additionally, NO_2_ molecules interact with oxygen adsorbed on the sensing surface if the sensor operates in the air:

|  | $2NO_{2 \left( \mathrm{gas} \right)}+ O_{2 (ads)}^{-}+ e^{-}\to2{NO}_{3 (ads)}^{-}.$ | (2) |
| --- | --- | --- |

Under UV light, oxygen photo ions participate in the reaction (2), and NO_2_ ions easily replace them, increasing the response.





**Figure S6** Time response (relative changes in sensor resistance) of MoS_2_ sensor to five cycles of NO_2_ of selected concentrations (1–10 ppm) introduction under UV light (275 nm) **after subtraction of drift baseline**. Each detection cycle consisted of 30 min-response and 30 min-recovery in S.A. The bias voltage was set to 20 V.

Figure S7 demonstrates the current-voltage characteristics of a test MoS_2_ sample as
a dark (solid curves) and UV-assisted (dashed curves) response to 5 ppm of NO_2_. The characteristics were measured after 15 minutes of exposure to the target gas. We calculated resistive responses at *V* = 20 V for each case to compare the sensitivity with and without light assistance. The resistive response was ~4 % for the dark case, whereas it increased around five times (to ~20 %) under UV light. This observation confirms the superiority of UV-assisted NO_2_ sensing. Additionally, the experiment confirmed the reproducibility of the gas sensing performance of the fabricated MoS_2_ sensors. Although the baseline resistance may differ between the particular samples due to the randomly distributed 2D flakes, the relative resistance changes under NO_2_ remained at the same level after 15 minutes of detection (19.7 % for the first sample and 20.4 % for the second sample).





**Figure S7** Current-voltage (*I*-*V*) characteristics of MoS_2_ sensor in S.A. and 5 ppm of NO_2_ in the dark (solid curves) and under UV light (275 nm) (dashed curves). Each detection cycle lasted 15 min. The current values at *V* = 20 V were extracted to calculate the resistive responses of the sensor.

Figure S8 presents the MoS_2_ sensor time response to selected concentrations of NH_3_ under UV irradiation (275 nm) after subtraction of the baseline drift. The sensor resistance decreases with increasing concentration of ammonia, which agrees with the reducing nature of this target gas. The donation of electrons by NH_3_ during adsorption can be formulated *via* the reaction:

|  | $4NH_{3 \left( \mathrm{gas} \right)}+ 5O_{2 (ads)}^{-}\to4NO+ {6H}_{2}O+ {5e}^{-}.$ | (3) |
| --- | --- | --- |

In this case, ammonia reacts with oxygen adsorbed on the surface, forming NO and H_2_O and releasing electrons to MoS_2_. Similarly to the NO_2_ case, photoinduced oxygen ions can react with ammonia, producing an analogous electron-donating effect.





**Figure S8** Time response (relative changes in sensor resistance) of MoS_2_ sensor to five cycles of NH_3_ of selected concentrations (2–12 ppm) introduction under UV light (275 nm) **after subtraction of drift baseline**. Each detection cycle consisted of 30 min-response and 30 min-recovery in S.A. The bias voltage was set to 20 V.

Figure S9 presents the MoS_2_ sensor time response to selected acetone concentrations under UV irradiation (275 nm) after subtracting the baseline drift. Similarly to NH_3_, the sensor resistance decreases with increasing acetone concentration, suggesting that this nucleophilic gas has reducing properties toward *n*-type MoS_2_. Compared to NO_2_ and NH_3_, the resistive responses to acetone are less stable, possibly due to the weak bonding of organic molecules to the sensing surface. The subtraction of the drifting baseline exposes the direction of charge transfer, which drives gas detection for MoS_2_ systems. Since the conductance of the sensor increases (resistance decreases), acetone adsorption is associated with electron transfer from the gas molecules to MoS_2_. As the resistive responses are lower for acetone than for NO_2_ or NH_3_, the electron transfer is presumably quantitatively lower for this organic gas. Acetone molecules react with the adsorbed oxygen as follows:

|  | $C_{3}H_{6}O_{(gas)}+ 4O_{2 (ads)}^{-}\to{3CO}_{2}+ {3H}_{2}O+ {4e}^{-}.$ | (4) |
| --- | --- | --- |

The adsorption is accompanied by the release of electrons to the conduction band of MoS_2_, causing a reducing effect similar to the one induced by NH_3_.





**Figure S9** Time response (relative changes in sensor resistance) of MoS_2_ sensor to five cycles of C_3_H_6_O of selected concentrations (2–12 ppm) introduction under UV light (275 nm) **after subtraction of drift baseline**. Each detection cycle consisted of 30 min-response and 30 min-recovery in S.A. The bias voltage was set to 20 V.

Figure S10 compares the time responsivity of the MoS_2_ sensor to 5 ppm of NO_2_, NH_3_, and C_3_H_6_O. Only oxidizing NO_2_ increases sensor resistance, whereas NH_3_ and C_3_H_6_O demonstrate reducing properties toward the investigated gas sensor. The DC resistance response to the same concentration of NO_2_ (0.25) is more than eight times higher than for NH_3_ (-0.03) and ~25 times higher than for acetone (-0.01). This shows the increased selectivity toward NO_2_ based on the DC responses only.

**

**

**Figure S10** Time response (relative changes in sensor resistance) of MoS_2_ sensor to the cycle of 5 ppm of NO_2_, NH_3_, and C_3_H_6_O introduction under UV light (275 nm). Each detection cycle consisted of 30 min-response and 30 min-recovery periods in S.A. The bias voltage was set to 20 V.

Figure S11 shows the response and recovery times derived from time-response curves after subtracting the drifting baseline for three investigated gases. The response and recovery times are single minutes and longer, but there is no strict correlation with the concentration of target gases. The point of 2 ppm for acetone was purposely omitted as such concentration of this gas provided the least readable and stable response even after subtracting the drifting baseline. We suppose that due to the time drift (the most intense in the cycles with lower concentrations sensing), the error connected with the estimation of the time constants may be a few times higher than the error coming from the resolution of the measurements.

**



**

**

**

**Figure S11** Response (red dots) and recovery times (blue squares) derived from the time-response curves measured for the MoS_2_ sensor for selected NO_2_, NH_3_, and C_3_H_6_O concentrations. The width of lines connecting the adjacent data points correspond to the error bars derived from the resolution of time measurements.

Figure S12 shows the effect of introducing relative humidity (RH) of 40% on the baseline resistance of the MoS_2_ sensor. The figure confirms that the humid air decreases the sensor resistance by 64%, but the sensor recovers to the original resistance in dry S.A. The effect on the baseline of the sensor is much more significant than on the responsivity to the selected target gases (see Table S2). To solve the issue of the baseline shift due to the variable humidity, using the MoS_2_ sensor in practical applications would benefit from normalizing the sensor baseline resistance and analytically correcting it so it reflects the dry conditions case.





**Figure S12** Time response (sensor resistance) of MoS_2_ sensor to introducing humid carrier gas of RH=40% under UV light (275 nm). The relative response to the humid air was 64%, according to the formula provided in the figure. The bias voltage was set to 20 V.

Table S2 compares MoS_2_ sensor responses toward 5 ppm of NO_2_, NH_3_, and C_3_H_6_O in dry and humid conditions. The responsivity to selected gases is maintained for four months after sensor fabrication, showing sensor stability in a few months period. Introducing humidity into the sensing system slightly changes the responses to all gases. The relative change in sensor response is only slightly visible in the case of NO_2_. However, it is more noticeable for NH_3_ and C_3_H_6_O, as these gases produce minor responses (1–3%) even in dry S.A. Interestingly, the sensor responsivity is less affected by surrounding humidity than the sensor baseline resistance, as demonstrated in Figure S12.

Table S2 Comparison of responses of the MoS_2_ sensor (relative changes in sensor resistance) to selected gases to show sensor stability in time (fresh *vs.* aged sample) and the effect of humidity on sensor sensitivity (dry S.A. and RH=40% case).

|  | (*R*_S_-*R*_0_)/*R*_0_ (%) | | |
| --- | --- | --- | --- |
|  | NO_2_ (5 ppm) | NH_3_ (5 ppm) | C_3_H_6_O (5 ppm) |
| Dry S.A.  (fresh sample) | 25.34 | 2.92 | 1.12 |
| Dry S.A.  (four months after fabrication) | 29.35 | 3.00 | 1.37 |
| Humid S.A.  (RH=40%) | 27.27 | 4.76 | 2.72 |

Figure S13 presents the time response to 5 ppm concentration of three target gases (NO_2_, NH_3_, C_3_H_6_O) at 60 °C and under continuous UV irradiation. Contrary to the sensor response at RT, elevated temperature suppresses the sensing properties toward target gases, resulting in constant time drift with a more unstable baseline. No characteristic detection cycles can be distinguished from the resistive time responses at 60 °C. Such phenomenon may be explained in a way that additional thermal energy applied to the sensing surface can disturb the equilibrium in surface processes, increasing the desorption rate and even detaching the target gas molecules. Moreover, randomly deposited MoS_2_ flakes can be subjected to structural changes, including migration and aggregation at constant, elevated temperatures, possibly resulting in changes in surface activity and induced resistance instability or drift. However, we want to note that a more detailed investigation should be completed to understand this phenomenon observed for our sensors. On the other hand, UV assistance at RT enabled efficient detection with low detection limits for inorganic (NO_2_, NH_3_) and organic (C_3_H_6_O) gases.

**

**

**Figure S13** Time response (relative changes in sensor resistance) of MoS_2_ sensor to subsequent cycles of 5 ppm of NO_2_, NH_3_, and C_3_H_6_O introduction under UV light (275 nm) and elevated temperature of **60 °C**. Each detection cycle consisted of 30 min-response and 30 min-recovery periods in S.A. The bias voltage was set to 20 V.

Table S3 summarizes the properties of MoS_2_-based gas sensors operating at RT and utilizing UV light assistance reported from 2019. The advantage of utilizing an ink-printed MoS_2_ sensor is the simplicity of its fabrication while still preserving the high sensitivity, specifically toward NO_2_ with DC response monitoring and NH_3_ with noise response monitoring. The selectivity of the ink-printed MoS_2_ sensor is enhanced with the FES method.

Table S3 Summary of reports on UV-assisted gas sensing by MoS_2_ at RT published from 2019.

| **Gas** | **Irradiation** | **Response*** | **DL** | **Comments** | **Year/Ref.** |
| --- | --- | --- | --- | --- | --- |
| NO_2_ | Red 660 nm | 50% (25 ppb) | 0.1 ppb | Nitrogen as a reference atmosphere | 2019/[1] |
| NO_2_ | UV 280 nm** | 25.3% (10 ppm) | - | UV light provided faster recovery than blue or white light | 2019/[2] |
| NO_2_ | Solar simulator | 20% (50 ppb) | 0.15 ppb | Response calculated based on current changes | 2021/[3] |
| NO_2_ | UV 254 nm** | ~70% (100 ppb) | 10 ppb | Response calculated based on current changes | 2023/[4] |
| **NO_2_** | **UV 275 nm** | **41% (10 ppm)** | **80 ppb** | **Ink-printed MoS_2_ flakes utilizing the FES method** | **This work** |
| NO | UV 254 nm | 72.45% (100 ppm) | - | Response under UV light higher than at temperatures up to 100 °C | 2019/[5] |
| NO | UV 365 nm | 60.25% (100 ppm) | - | Response under UV light higher than at temperatures up to 100 °C | 2019/[5] |
| NO | UV 365 nm | 200% (60 ppb) | - | Cone-shaped MoS_2_ bilayer | 2019/[6] |
| NO | White light | 75% (60 ppb) | - | Cone-shaped MoS_2_ bilayer | 2019/[6] |
| NH_3_ | UV 280 nm** | ~2% (10 ppm) | - | UV light provided faster recovery than blue or white light | 2019/[2] |
| NH_3_ | UV 254 nm** | ~1% (2 ppm) | 500 ppb | Response calculated based on current changes | 2023/[4] |
| **NH_3_** | **UV 275 nm** | **24% (10 ppm)** | **130 ppb** | **Ink-printed MoS_2_ flakes utilizing the FES method** | **This work** |
| **C_3_H_6_O** | **UV 275 nm** | **16% (10 ppm)** | **360 ppb** | **Ink-printed MoS_2_ flakes utilizing the FES method** | **This work** |

*Response = the absolute value of the relative change in the sensor resistance in reference to air/inert atmosphere unless indicated otherwise in the comments

**UV irradiation applied only for the recovery phase

[1] T. Pham, G. Li, E. Bekyarova, M.E. Itkis, A. Mulchandani, MoS_2_-Based Optoelectronic Gas Sensor with Sub-parts-per-billion Limit of NO_2_ Gas Detection, ACS Nano. 13 (2019) 3196–3205. https://doi.org/10.1021/acsnano.8b08778.

[2] Y. Kang, S. Pyo, E. Jo, J. Kim, Light-assisted recovery of reacted MoS_2_ for reversible NO_2_ sensing at room temperature, Nanotechnology. 30 (2019) 355504. https://doi.org/10.1088/1361-6528/ab2277.

[3] H. Tabata, H. Matsuyama, T. Goto, O. Kubo, M. Katayama, Visible-Light-Activated Response Originating from Carrier-Mobility Modulation of NO2 Gas Sensors Based on MoS_2_ Monolayers, ACS Nano. 15 (2021) 2542–2553. https://doi.org/10.1021/acsnano.0c06996.

[4] M. Chen, D. Cui, N. Wang, S. Weng, Z. Zhao, F. Tian, X. Gao, K. He, C.-T. Chiang, S. Albawardi, S. Alsaggaf, G. Aljalham, M.R. Amer, C. Zhou, Inkjet-Printed MoS_2_ Nanoplates on Flexible Substrates for High-Performance Field Effect Transistors and Gas Sensing Applications, ACS Appl. Nano Mater. 6 (2023) 3236–3244. https://doi.org/10.1021/acsanm.2c04885.

[5] S. Ramu, T. Chandrakalavathi, G. Murali, K.S. Kumar, A. Sudharani, M. Ramanadha, K.R. Peta, R. Jeyalakshmi, R.P. Vijayalakshmi, UV enhanced NO gas sensing properties of the MoS2 monolayer gas sensor, Mater. Res. Express. 6 (2019). https://doi.org/10.1088/2053-1591/ab20b7.

[6] Y.Z. Chen, S.W. Wang, C.C. Yang, C.H. Chung, Y.C. Wang, S.W. Huang Chen, C.W. Chen, T.Y. Su, H.N. Lin, H.C. Kuo, Y.L. Chueh, An indoor light-activated 3D cone-shaped MoS_2_ bilayer-based NO gas sensor with ppb-level detection at room-temperature, Nanoscale. 11 (2019) 10410–10419. https://doi.org/10.1039/c8nr10157d.
